# Prediction Models for Postoperative Delirium of Cardiovascular Surgery (PODOCVS): Protocol for a Systematic Review

**DOI:** 10.2196/75368

**Published:** 2025-06-09

**Authors:** Xuling Zhao, Yike Wang, Liju Li, Meijuan Lan, Xiaodi He

**Affiliations:** 1 Zhejiang Taizhou Hospital Linhai China; 2 The Second Affiliated Hospital of Zhejiang University School of Medicine Hangzhou China

**Keywords:** prediction models, postoperative delirium, cardiovascular surgery, acute brain dysfunction, machine learning, systematic review

## Abstract

**Background:**

Postoperative delirium of cardiovascular surgery (PODOCVS) is an acute brain dysfunction characterized by inattention, impaired consciousness, and cognitive disorders, and the severity and presence of these symptoms fluctuate over time. PODOCVS occurs during the early postoperative period and is associated with adverse outcomes, including prolonged mechanical ventilation, premature mortality, and so on. Advances in its early diagnosis and treatment have mitigated some of the initial adverse effects of PODOCVS, but models for predicting risk in patients who have already developed PODOCVS remain inadequate for effective secondary prevention. Developing multivariable prediction models for stratifying PODOCVS risk would enable early, personalized interventions.

**Objective:**

This study aims to systematically review and critically evaluate the development, performance, and applicability of existing prediction models for PODOCVS.

**Methods:**

An extensive systematic search will be performed across multiple databases, including Embase, PubMed, the Web of Science, and so on, to identify studies related to multivariate predictive models for PODOCVS. A manual search of the included studies’ reference lists will also be conducted to identify any additional relevant publications. This systematic review will include studies that meet the following criteria: (1) studies with subject populations comprising adult cardiovascular surgery patients aged ≥18 years, (2) studies involving the development and internal or external validation of predictive models for PODOCVS via multivariate analysis, and (3) studies with outcome measures focused on postoperative delirium. Two researchers (ZXL and WYK) will independently extract the data and assess the included studies’ model quality using the Critical Appraisal and Data Extraction for Systematic Reviews of Prediction Modelling Studies (CHARMS) checklist and the Predictive Model Bias Risk Assessment Tool (PROBAST). Since this study will not involve patient data, ethics approval is not required. Our findings will be published in a peer-reviewed scientific journal and the dataset will be made freely available.

**Results:**

Literature searches were conducted from the inception of the database to May 20, 2024 (updated up to January 31, 2025), and data extraction and analysis are expected to be complete by the end of May 2025. We currently have a preliminary plan to publish the complete study results by August 2025, subject to any unforeseen delays or changes in the research timeline.

**Conclusions:**

We present a protocol for the systematic review of prediction models for postoperative delirium in cardiac surgery patients. Aiming to identify, summarize, and critically appraise existing risk models globally, this review seeks to provide an up-to-date reference for stakeholders involved in patients with cardiac surgery care, policy making, and research. In addition, we aim to investigate whether machine learning models for PODOCVS offer more accurate predictions than traditional statistical models.

**Trial Registration:**

PROSPERO CRD42024578957; https://www.crd.york.ac.uk/PROSPERO/view/CRD42024578957

**International Registered Report Identifier (IRRID):**

DERR1-10.2196/75368

## Introduction

### Background

Postoperative delirium of cardiovascular surgery (PODOCVS) is an acute brain dysfunction characterized by inattention, impaired consciousness, and cognitive impairment that fluctuates in presence and severity across time [1].

Studies show that PODOCVS affects approximately 26% to 52% of patients with cardiovascular surgery [2]. As a common complication of cardiovascular surgery, PODOCVS worsens patient outcomes [3] and consumes enormous financial resources in excess of US $164 billion per year [4]. PODOCVS is independently associated with major surgical trauma, advanced age, cardiopulmonary bypass, compromised brain health, and transfer to the cardiovascular intensive care unit post surgery [2,5,6]. PODOCVS occurs during the early postoperative period and is associated with adverse outcomes including prolonged mechanical ventilation times and hospital stays, long-term nursing needs, worsening of pre-existing cognitive impairment, new dementia, increased anxiety and depression, and premature mortality [7]. In terms of pharmacological therapies, ramelteon and suvorexant can reportedly reduce delirium, but premedication to prevent delirium is not recommended for all patients [8]. However, due to the lack of specific treatments, strategies for managing delirium currently focus only on its prevention and early detection [9,10]. Therefore, identifying populations at high risk of developing delirium and providing targeted and effective interventions are crucial [9,11]. Risk stratification and identification of vulnerable patients offer an extremely efficient method of protecting them from the initial adverse outcomes of PODOCVS. The pathogenesis of PODOCVS is multifactorial and, as such, multivariable prediction models for stratifying PODOCVS risk may enable early personalized treatment interventions [12-14]. This would significantly reduce the incidence of delirium, shorten hospital stays, cut medical costs, and reduce the incidence of postoperative complications and mortality risk [4].

At present, there are many PODOCVS prediction models used in clinical practice. These can be divided into traditional statistical models and machine learning (ML) models. Although statistical models (eg, logistic regression) are favorable in terms of model interpretability, Choi et al [15] indicate that ML is preferred for predictive accuracy. The predictive power of traditional statistics-based models (eg, the E-PRE-DELIRIC [Early Prediction Model for Delirium in the intensive care unit] model) varies greatly across regional cohorts with an area under the curve (AUC) of 0.54, which is below the receiver operating characteristic curve of 0.75 [16,17]. Mufti et al [18] and Xue et al [19] have shown that the predictive accuracy of ML algorithms was superior to that of conventional statistical models in the area of PODOCVS [20]. ML models can handle complex, multidimensional data without the limitations of traditional statistical methods. Although there is an expanding body of published literature on the use of ML models in cardiovascular health care, whether their performance in predicting PODOCVS is superior to that of traditional statistical models remains to be verified [21].

The accuracy of predictive models for patients at risk of developing delirium has been reported to be insufficient when applied to patients with cardiac surgery [17]. Hence, predictive models for delirium risk should be constructed for patients with cardiac surgery specifically [22]. In recent years, an increasing number of studies have focused on developing or validating predictive models to estimate PODOCVS risk. However, health care professionals are uncertain as to which model to use for patients undergoing cardiac surgery in specific patterns, settings, and populations. Hence, the quality of existing models and their applicability remain unclear. Therefore, it is critical to thoroughly evaluate the predictive performance, applicability, and quality of existing PODOCVS risk prediction models [23]. To this end, this study presents a protocol for the systematic review and critical assessment of the quality, performance, and applicability of current predictive models for PODOCVS risk.

### Objectives

The systematic review aims to thoroughly evaluate the predictive performance, applicability, and quality of existing PODOCVS risk prediction models. To provide a robust scientific foundation for health care professionals to select appropriate delirium prediction models tailored to patients with cardiac surgery in specific clinical environments. The objectives of this project are listed in Textbox 1.

**Research aims.**

- The construction methodology and model validation approach of the existing postoperative delirium of cardiovascular surgery risk prediction model were thoroughly analyzed, and the predictors were ranked based on the prevalence and strength of the predictors.
- Comprehensively assess the predictive performance, applicability, and quality of existing postoperative delirium of cardiovascular surgery risk prediction models.
- Inform stakeholders, such as policy makers and health care workers directly involved in the treatment of patients with cardiac surgery, on the available delirium prediction models and their setting-specific clinical utility, strengths, and limitations.
- Determine whether postoperative delirium of cardiovascular surgery machine learning models can provide more accurate predictions as compared with postoperative delirium of cardiovascular surgery traditional statistical models.

## Methods

### Study Design and Data Source

This protocol was developed according to the PRISMA-P (Preferred Reporting Items for Systematic Reviews and Meta-Analyses Protocols) guidelines (Multimedia Appendix 1) [24].

A systematic review will be conducted according to the recommended methods for systematic reviews and meta-analyses of prediction models [24] and will adhere to the CHARMS (Critical Appraisal and Data Extraction for Systematic Reviews of Prediction Modeling Studies) checklist [25]. The results of this review will be reported as per the updated PRISMA (Preferred Reporting Items for Systematic Reviews and Meta-Analyses) 2020 checklist [26]. The review will be registered in the International PROSPERO (International Prospective Register of Systematic Reviews). Any modifications to the protocol will be amended accordingly.

### Eligibility Criteria

This review follows the PICOTS (Population, Intervention model, Comparator, Outcomes, Timing, and Setting) approach to frame the review question and determine the inclusion criteria (Table 1) [25,26].

This systematic review will include studies with a subject population of patients with cardiac surgery aged 18 years and older, and studies using developed and internally or externally validated predictive models for PODOCVS risk based on multivariate analysis. The outcome metric is postoperative delirium. Studies will not be excluded based on their publication status and language. The exclusion criteria are as follows: (1) studies focused solely on the external validation of existing models; (2) studies that construct predictive models without conducting internal/external verification; (3) studies for which data are unavailable through the accessible channels; (4) studies lacking evidence of mode performance in derivation or validation cohorts; (4) studies that only analyze predictive risk factors without constructing predictive models; and (5) various types of publications such as conference abstracts, reviews, comments, research protocols, animal model studies, and textbook materials.

**Table 1:** Eligibility criteria are based on the Population, Intervention model, Comparator, Outcomes, Timing, and Setting.

	Criteria
Population	The population of interest comprises patients with cardiac surgery aged 18 years and older.
Index model	This review will include studies involving the development and concurrent internal or external validation of predictive models for postoperative delirium of cardiovascular surgery risk. Studies that focus exclusively on the external validation of existing models and studies that construct predictive models without conducting internal/external validation will be excluded from consideration.
Comparator	Not applicable.
Outcome	The outcome is defined as postoperative delirium.
Timing	Outcomes will be predicted using either preoperative, intraoperative, or postoperative conditions.
Setting	Predictive models are intended to perform risk stratification for the development of delirium, enabling the implementation of preventive measures and early intervention.

### Search Strategy

Once this protocol is published, the search will be performed in the following databases: Embase, PubMed, the Web of Science Core Collection, the China National Knowledge Infrastructure (CNKI), Wan Fang, and Wei Pu (VIP). The following search terms will be used: “cardiac surgical procedure,” “cardiac surgery,” “cardiovascular surgery,” “heart surgery,” “coronary artery bypass grafting,” “Coronary Artery Bypass Grafting (CABG),” “extracorporeal circulation,” “type A aortic dissection,” “type B aortic dissection,” “subacute delirium,” “delirium,” “postoperative delirium,” “delirium of mixed origin,” “deliri*,” “psychosis,” “intensive care delirium,” “neurological complications,” “risk prediction,” “model,” “risk score,” and “risk assessment”. The retrieval time limit will range from each corresponding database’s inception until May 20, 2024 (updated until January 31, 2025). The search will be performed by 2 authors (WYK and ZXL). We will perform both forward and backward citation searches for the included studies and relevant previous systematic reviews. The complete search strings are presented in Multimedia Appendix 2.

### Selection Process

EndNote’s “Delete duplicates” function will be used, followed by the manual deletion of any remaining duplicates. Subsequently, the authors (ZXL and WYK) will evaluate the acceptability of the titles and abstracts. In case of an eventual disagreement between the authors who analyze the eligibility of the documents, the opinion of a third author (LLJ) will be considered. Any inconsistencies will be resolved through discussion to arrive at a consensus. Thereafter, the selected full texts will finally be included in the review.

### Data Extraction and Management

The following information will be identified from each selected study and summarized in tables for qualitative analysis: author name, country, the aim of the study, model development sample size, external validation sample size, internal validation sample size, participants, follow-up, study design (prospective study or retrospective study), events per variable, the main outcome, predictors in the final model, modeling methodology, number of models, model characteristics (ie, modeling methods, model validation methods, and candidate predictors) and model performances (ie, discrimination, calibration, and classification measures; Table 2). Data will be extracted independently by 2 authors (ZXL and WYK), who will enter the data into different Microsoft Excel spreadsheets. A third author will verify their agreement and resolve disagreements by reanalyzing the data (LLJ). A further limitation of the planned review is that we will not contact study authors to request unreported information, as we will explicitly document instances where information is missing.

**Table 2:** Information for data extraction and subsequent summary and appraisal. Adapted from Checklist for Critical Appraisal and Data Extraction for Systematic Reviews of Prediction Modelling Studies and Prediction model Risk Of Bias Assessment Tool.

Domain	Key items
Source of data	Source of data (eg, cohort, case-control, randomized trial participants, registry data, etc).
Participants	Participant eligibility and recruitment method (eg, location, number of centers, setting, and inclusion and exclusion criteria). Participant description (age, sex, Types of cardiovascular surgery, and postoperative follow-up time). Details of undergoing cardiovascular surgery. How delirium diagnosis is defined (whether consistent for all participants, clinical history and physical signs, etc). Study dates.
Outcomes to be predicted	Type of outcome (eg, single or combined endpoints). Definition and method for measurement of outcome (delirium and subtypes of delirium). Was the same outcome definition (and measurement method) used in all patients? Time of outcome occurrence or summary of duration of follow-up. Was the outcome assessed without knowledge of the candidate predictors (ie, blinded)?
Candidate predictors	Number and type of predictors (eg, demographics, patient history, physical examination, laboratory parameters, etc). Definition and method for measurement of candidate predictors (including whether defined and measured in a similar way for all participants). Timing of predictor measurement (eg, at patient presentation, at diagnosis, at treatment initiation, or otherwise). Handling of predictors in the modeling (eg, continuous, linear, nonlinear transformations or categorized).
Sample size	Number of participants and number of outcomes. Events per candidate predictor. Whether the authors describe a sample size calculation.
Missing data	Number of participants with any missing value (including predictors and outcomes). Number of participants with missing data for each predictor. Handling of missing data (eg, complete-case analysis, imputation, or other methods).
Model development	Modeling method (eg, logistics, survival, or other). Modeling assumptions satisfied. Description of participants that were excluded from the analysis with justification. Method for selection of predictors for inclusion in multivariable modeling (eg, all candidate predictors, preselection based on unadjusted association with the outcome). Method for selection of predictors during multivariable modeling (eg, full model approach, backward or forward selection) and criteria used (eg, *P* value, Akaike information criterion). Shrinkage of predictor weights or regression coefficients (eg, no shrinkage, uniform shrinkage, penalized estimation).
Model performance	Calibration (calibration plot, calibration slope, Hosmer-Lemeshow test), discrimination (C-statistic, D-statistic, and log-rank), and overall performance measures with CIs. Classification measures (eg, sensitivity, specificity, predictive values, and net reclassification improvement) and whether a priori cutoff points were used.
Model evaluation	Method used for testing model performance: development dataset only (apparent performance, a random split of data, resampling methods, for example, bootstrap or cross-validation or none) or separate external validation. For external validations; data source and participants to be described as per “source of data” and “participants” domains. Definitions and distributions (including missing data) of outcome and candidate predictors.
Traditional prediction model	Traditional algorithm-based prediction models refer to those that use classical statistical methods for forecasting future events or values, for example, linear regression, and logistic regression.
Machine learning prediction model	Machine learning–based prediction models are capable of processing and analyzing extensive historical datasets, extracting underlying patterns and rules, and leveraging the acquired knowledge to forecast future scenarios, for example, extreme gradient boosting, support vector machine, adaptive boosting, multilayer perceptron, neural network, naive Bayes, and gradient boosting machine. In case of poor external validation, whether the model was updated or extended (eg, intercept recalibrated, predictor effects adjusted, or new predictors added).
Results	Final and other multivariable models presented, including predictor weights or regression coefficients, intercept, baseline survival, and model performance measures (with standard errors or CIs). Any alternative presentation of the final prediction models, for example, sum score, nomogram, score chart, and predictions for specific risk subgroups with performance. Comparison of the definition and distribution of predictors (including missing data) for development and validation datasets.
Interpretation and discussion	Study authors’ interpretation of presented models (intended use and clinical utility, etc). Study authors’ reported strengths and limitations.

### Risk of Bias in Individual Studies

Two authors (ZXL and WYK) will independently use the CHARMS checklist [25] to extract the relevant key information. Candidate studies with uncertain model characteristics (ie, modeling methods, model validation methods, and candidate predictors) and model performances (ie, discrimination, calibration, and classification measures) will be discussed with a third author (LLJ) before inclusion.

We will use the Prediction Model Risk of Bias Assessment Tool (PROBAST) to evaluate the quality of the candidate studies, as evaluated independently by 2 authors (ZXL and WYK). Similarly, uncertain cases will be discussed with a third author (LLJ) before inclusion. PROBAST [27] comprises 4 domains, namely, participants, predictors, outcome, and analysis. Each domain includes 20 signaling questions used to evaluate the risk of bias and applicability. These signaling questions rely on factual information and are categorized as either “Yes” or “Probably yes” (Y), “No” or “Probably no” (N), or “No information” (NI). After evaluating each domain using signaling questions specific to that domain, they will be categorized as either “High,” “Low,” or “Unclear.” By synthesizing the results obtained from each dimension, an overall assessment will be made regarding the predictive models’ risk of bias and applicability.

### Assessing the Models’ Predictive Performance and Accuracy

The evaluation of the predictive models’ performance will be based on an assessment of their discrimination and calibration [28]. “Discrimination” refers to a model’s ability to distinguish between individuals with different outcomes or conditions. It assesses how well the model correctly ranks individuals based on their risk of experiencing a certain event or condition. Common methods for evaluating discrimination include the c-statistic (also referred to as the area under the receiver operating characteristic curve) and the concordance index [28]. “Calibration” refers to the conformity between a model’s predicted probabilities or risk evaluations and the observed actual outcomes. Calibration is often assessed by comparing the predicted and observed event rates among different risk groups, or by using calibration plots [20,28].

### Ethical Considerations

Ethics approval is not required for this systematic review, as it does not require primary data collection. The protocol will be registered with the PROSPERO. The results of this systematic review will be disseminated through publication in an academic journal and scientific conferences.

## Results

Literature searches were conducted from the inception of the database to May 20, 2024 (updated up to January 31, 2025), and data extraction and analysis are expected to be complete by the end of May 2025. We currently have a preliminary plan to publish the complete study results by August 2025, subject to any unforeseen delays or changes in the research timeline. The findings will be used to inform stakeholders, including policy makers and health care professionals directly involved in the treatment of patients with cardiac surgery, about the existing delirium prediction models, their setting-specific clinical utility, strengths, and limitations. In addition, it serves as a reference for selecting between traditional statistical models and ML models in future research.

## Discussion

### Expected Results and Practical Implications

This protocol aims to provide a detailed description of the process for conducting a systematic review of existing global predictive models for PODOCVS, focusing on their predictive performance, applicability, and quality. Due to differences in the basic characteristics of study populations, protocols, surgical methods, and evaluation methods and frequency, the reported incidence of PODOCVS varies greatly [29-31]. PODOCVS can cause serious adverse outcomes [32], and prevention is the most effective strategy for minimizing its occurrence and poor prognosis [2]. Accurate risk estimation for surgical patients using predictive models can aid clinical decision-making and inform policy, thereby guiding the optimal allocation of often-limited resources. By identifying, summarizing, and evaluating published PODOCVS prediction models, this systematic review will serve as a comprehensive resource for PODOCVS stakeholders, including health care workers, policy makers, and researchers. Although numerous PODOCVS prediction models exist, most of them have not been developed, validated, and assessed according to established guidelines for predictive research. This has given rise to significant biases in PODOCVS risk estimation, deficiencies in the statistical methods used, and a lack of internal and external validation.

While the clinical outcomes of PODOCVS are heterogeneous, many predictors of poor clinical outcomes have been identified. These include age (≥60 years old), sex (male), education level, history of living alone, frailty, BMI (>30 kg/m^2^), high EuroSCORE (≥14), disease severity, changes in lifestyle patterns before and after admission, personality traits (high irritability), and history of smoking and alcohol consumption [33-35]. These interrelated factors’ relative influence on patient outcomes can be described through multivariable modeling and subsequently used to construct a predictive model to estimate patients with cardiac surgery risk of developing postoperative delirium [36]. Therefore, to ensure the validity of the included predictive models, we will only select models that are internally or externally validated during development. Studies focused solely on the external validation of existing models or otherwise unvalidated predictive models will be excluded since several existing externally validated models have exhibited significant variability in their predictive accuracy across different populations. The reason for this may be that postoperative delirium in patients with cardiac surgery is closely related to the type of surgery that patients receive [2,37]. The planned systematic review will concisely summarize key information presented across all identified predictive model studies. Patients with cardiac surgery health care providers and policy makers can then use this information to assess a model’s applicability to the patients with cardiac surgery population in different scenarios.

In recent years, ML-based predictive models have shown great success [38]. ML models can process vast quantities of multidimensional and unstructured data to reveal the roles and relationships between multiple variables, providing an effective means for accurately predicting delirium risk in patients with cardiac surgery [9,39,40]. Although ML-based predictive models exhibit good predictive performance, research has shown that they lack explanatory power. The reason for this may be that patients’ clinical data are not uniform and standardized, and key data are often omitted [41]. Which of the 2 model types (ie, ML-based or statistical models) exhibits superior discrimination and calibration in the prediction of PODOCVS risk has not been demonstrated. To some extent, this affects the selection and use of predictive models by clinical medical staff. Therefore, this review will also analyze whether ML models can provide more accurate predictions as compared with traditional statistical models.

Strengths of this study include its strict adherence to the updated PRISMA 2020 checklist and its planned adherence to the CHARMS checklist. The use of a consensus-based analytical approach fosters collective accountability in interpretative decisions, thereby strengthening the robustness and credibility of the findings.

### Limitations

Some limitations of the study warrant consideration. We will only conduct qualitative descriptions and not perform a meta-analysis. We anticipate that inconsistencies in the predictors included in the models, follow-up duration, clinical settings in which the models were developed, types of cardiovascular surgery, and geographic distribution of the study populations may pose challenges to conducting a meta-analysis. Even if each predictive model we included has undergone internal or external validation, its limited clinical application necessitates further evaluation of its generalizability to ensure both applicability and scientific rigor. Although the study uses a comprehensive methodology, one limitation is the omission of a search for grey literature, which could potentially result in the exclusion of studies reported in nontraditional publications. Another limitation of the planned review is that we will not contact study authors to request unreported data, although missing information will be explicitly documented.

### Conclusion

We present a protocol for the systematic review of prediction models of postoperative delirium for patients with cardiac surgery. With the aim of identifying, summarizing, and appraising the available risk models, we hope to provide a current reference to stakeholders engaged in patients with cardiac surgery care, policy, and research. In addition, we also hope to explore whether PODOCVS ML models can provide more accurate predictions as compared with PODOCVS traditional statistical models.

## Appendix

Multimedia Appendix 1 PRISMA-P checklist.

Multimedia Appendix 2 Search strategies.

## Abbreviations

AUC: area under the curve
CHARMS: Critical Appraisal and Data Extraction for Systematic Reviews of Prediction Modelling Studies
CNKI: China National Knowledge Infrastructure
E-PRE-DELIRIC: Early Prediction Model for Delirium in the intensive care unit
ML: machine learning
PICOTS: Population, Intervention model, Comparator, Outcomes, Timing, and Setting
PODOCVS: postoperative delirium of cardiovascular surgery
PRISMA: Preferred Reporting Items for Systematic Reviews and Meta-Analyses
PRISMA-P: Preferred Reporting Items for Systematic Reviews and Meta-Analyses Protocols)
PROBAST: Predictive Model Bias Risk Assessment Tool
PROSPERO: International Prospective Register of Systematic Reviews

## References

[1] Cohen CL, Atkins KJ, Evered LA, Silbert BS, Scott DA (2023). Examining subjective psychological experiences of postoperative delirium in older cardiac surgery patients. Anesth Analg.

[2] Cai S, Li J, Gao J, Pan W, Zhang Y (2022). Prediction models for postoperative delirium after cardiac surgery: Systematic review and critical appraisal. Int J Nurs Stud.

[3] Sugimura Y, Sipahi NF, Mehdiani A, Petrov G, Awe M, Minol JP, Boeken U, Korbmacher B, Lichtenberg A, Dalyanoglu H (2020). Risk and consequences of postoperative delirium in cardiac surgery. Thorac Cardiovasc Surg.

[4] Zhang Y, Wan D, Chen M, Li Y, Ying H, Yao G, Liu Z, Zhang G (2023). Automated machine learning-based model for the prediction of delirium in patients after surgery for degenerative spinal disease. CNS Neurosci Ther.

[5] Lin J, Zheng G, Chen L, Luo Z (2023). A nomogram model for assessing predictors and prognosis of postoperative delirium in patients receiving acute type A aortic dissection surgery. BMC Cardiovasc Disord.

[6] Eremenko AA, Chemova EV (2014). [Comparison of dexmedetomidine and propofol for short-term sedation in early postoperative period after cardiac surgery]. Anesteziol Reanimatol.

[7] Yokoyama C, Yoshitnai K, Ogata S, Fukushima S, Matsuda H (2023). Effect of postoperative delirium after cardiovascular surgery on 5-year mortality. JA Clin Rep.

[8] Nagata C, Hata M, Miyazaki Y, Masuda H, Wada T, Kimura T, Fujii M, Sakurai Y, Matsubara Y, Yoshida K, Miyagawa S, Ikeda M, Ueno T (2023). Development of postoperative delirium prediction models in patients undergoing cardiovascular surgery using machine learning algorithms. Sci Rep.

[9] Han C, Kim HI, Soh S, Choi JW, Song JW, Yoon D (2024). Machine learning with clinical and intraoperative biosignal data for predicting postoperative delirium after cardiac surgery. iScience.

[10] Jin Z, Hu J, Ma D (2020). Postoperative delirium: perioperative assessment, risk reduction, and management. Br J Anaesth.

[11] Curtis MS, Forman NA, Donovan AL, Whitlock EL (2020). Postoperative delirium: why, what, and how to confront it at your institution. Curr Opin Anaesthesiol.

[12] Yang T, Yang H, Liu Y, Liu X, Ding Y, Li R, Mao A, Huang Y, Li X, Zhang Y, Yu F (2024). Postoperative delirium prediction after cardiac surgery using machine learning models. Comput Biol Med.

[13] Sadlonova M, Hansen N, Esselmann H, Celano CM, Derad C, Asendorf T, Chebbok M, Heinemann S, Wiesent A, Schmitz J, Bauer FE, Ehrentraut J, Kutschka I, Wiltfang J, Baraki H, von Arnim CA, FINDERI investigators (2024). Preoperative delirium risk screening in patients undergoing a cardiac surgery: Results from the prospective observational FINDERI study. Am J Geriatr Psychiatry.

[14] Zhao X, Li J, Xie X, Fang Z, Feng Y, Zhong Y, Chen C, Huang K, Ge C, Shi H, Si Y, Zou J (2024). Online interpretable dynamic prediction models for postoperative delirium after cardiac surgery under cardiopulmonary bypass developed based on machine learning algorithms: A retrospective cohort study. J Psychosom Res.

[15] Choi SG, Oh M, Park D, Lee B, Lee Y, Jee SH, Jeon JY (2023). Comparisons of the prediction models for undiagnosed diabetes between machine learning versus traditional statistical methods. Sci Rep.

[16] Gao W, Zhang Y, Jin J (2022). Validation of E-PRE-DELIRIC in cardiac surgical ICU delirium: A retrospective cohort study. Nurs Crit Care.

[17] Lee A, Mu J, Joynt G, Chiu C, Lai V, Gin T, Underwood M (2017). Risk prediction models for delirium in the intensive care unit after cardiac surgery: a systematic review and independent external validation. Br J Anaesth.

[18] Mufti HN, Hirsch GM, Abidi SR, Abidi SSR (2019). Exploiting machine learning algorithms and methods for the prediction of agitated delirium after cardiac surgery: Models development and validation study. JMIR Med Inform.

[19] Xue X, Chen W, Chen X (2022). A novel radiomics-based machine learning framework for prediction of acute kidney injury-related delirium in patients who underwent cardiovascular surgery. Comput Math Methods Med.

[20] Kanwar MK, Kilic A, Mehra MR (2021). Machine learning, artificial intelligence and mechanical circulatory support: A primer for clinicians. J Heart Lung Transplant.

[21] Christodoulou E, Ma J, Collins GS, Steyerberg EW, Verbakel JY, Van Calster B (2019). A systematic review shows no performance benefit of machine learning over logistic regression for clinical prediction models. J Clin Epidemiol.

[22] Lindroth H, Bratzke L, Purvis S, Brown R, Coburn M, Mrkobrada M, Chan MTV, Davis DHJ, Pandharipande P, Carlsson CM, Sanders RD (2018). Systematic review of prediction models for delirium in the older adult inpatient. BMJ Open.

[23] 24 Jia Y Y (2024). A systematic review of prognostic prediction models for patients with chronic heart failure in China J. Chinese Journal of Thoracic and Cardiovascular Surgery Clinical Edition.

[24] Moher D, Shamseer L, Clarke M, Ghersi D, Liberati A, Petticrew M, Shekelle P, Stewart LA, PRISMA-P Group (2015). Preferred reporting items for systematic review and meta-analysis protocols (PRISMA-P) 2015 statement. Syst Rev.

[25] Moons KGM, de Groot JAH, Bouwmeester W, Vergouwe Y, Mallett S, Altman DG, Reitsma JB, Collins GS (2014). Critical appraisal and data extraction for systematic reviews of prediction modelling studies: the CHARMS checklist. PLoS Med.

[26] Debray TPA, Damen JAAG, Snell KIE, Ensor J, Hooft L, Reitsma JB, Riley RD, Moons KGM (2017). A guide to systematic review and meta-analysis of prediction model performance. BMJ.

[27] Wolff RF, Moons KG, Riley RD, Whiting PF, Westwood M, Collins GS, Reitsma JB, Kleijnen J, Mallett S (2019). PROBAST: A tool to assess the risk of bias and applicability of prediction model studies. Ann Intern Med.

[28] Damen JAA, Moons KGM, van Smeden M, Hooft L (2023). How to conduct a systematic review and meta-analysis of prognostic model studies. Clin Microbiol Infect.

[29] Plaschke K, Fichtenkamm P, Schramm C, Hauth S, Martin E, Verch M, Karck M, Kopitz J (2010). Early postoperative delirium after open-heart cardiac surgery is associated with decreased bispectral EEG and increased cortisol and interleukin-6. Intensive Care Med.

[30] Schoen J, Meyerrose J, Paarmann H, Heringlake M, Hueppe M, Berger K (2011). Preoperative regional cerebral oxygen saturation is a predictor of postoperative delirium in on-pump cardiac surgery patients: a prospective observational trial. Crit Care.

[31] Brown CH (2014). Delirium in the cardiac surgical ICU. Curr Opin Anaesthesiol.

[32] Wang Y, Zhang C (2024). Incidence of postoperative delirium and its modifiable risk factors in patients undergoing cardiac surgery: A prospective observational study based on propensity score matching. Medical Journal of Chinese People's Liberation Army.

[33] Huang W, Wu Q, Zhang Y, Tian C, Huang H, Wang H, Mao J (2022). Development and validation of a nomogram to predict postoperative delirium in type B aortic dissection patients underwent thoracic endovascular aortic repair. Front Surg.

[34] Greaves D, Psaltis PJ, Davis DHJ, Ross TJ, Ghezzi ES, Lampit A, Smith AE, Keage HAD (2020). Risk factors for delirium and cognitive decline following coronary artery bypass grafting surgery: A systematic review and meta‐analysis. J Am Heart Assoc.

[35] Tan C, Saito N, Miyawaki I, Shiotani H (2020). Preoperative circadian physical activity rhythm and postoperative delirium in cardiovascular surgery patients. Chronobiol Int.

[36] Wilson J, Chowdhury F, Hassan S, Harriss EK, Alves F, Dahal P, Stepniewska K, Guérin PJ (2023). Prognostic prediction models for clinical outcomes in patients diagnosed with visceral leishmaniasis: protocol for a systematic review. BMJ Open.

[37] Cardiac Critical Care Branch of China International Exchange Promotive Association for Medical Health (2023). [Chinese expert consensus on the prevention and treatment of postoperative delirium of cardiovascular surgery]. Chin J Med.

[38] Suliman A, Masud M, Serhani MA, Abdullahi AS, Oulhaj A (2024). Predictive performance of machine learning compared to statistical methods in time-to-event analysis of cardiovascular disease: a systematic review protocol. BMJ Open.

[39] Luo W, Phung D, Tran T, Gupta S, Rana S, Karmakar C, Shilton A, Yearwood J, Dimitrova N, Ho TB, Venkatesh S, Berk M (2016). Guidelines for developing and reporting machine learning predictive models in biomedical research: A multidisciplinary view. J Med Internet Res.

[40] Zhao X, Li J, Xie X, Fang Z, Feng Y, Zhong Y, Chen C, Huang K, Ge C, Shi H, Si Y, Zou J (2024). Online interpretable dynamic prediction models for postoperative delirium after cardiac surgery under cardiopulmonary bypass developed based on machine learning algorithms: A retrospective cohort study. J Psychosom Res.

[41] Wang S, Zhu X (2022). Predictive modeling of hospital readmission: Challenges and solutions. IEEE/ACM Trans Comput Biol and Bioinf.

