# Supplementary material for: Prediction Models for Postoperative Delirium of Cardiovascular Surgery (PODOCVS): Protocol for a Systematic Review
Source: JMIR Res Protoc. 2025 Jun 9;14:e75368. doi: 10.2196/75368 (PMC12186001; doi:10.2196/75368)
Supplement: Multimedia Appendix 2 [file resprot_v14i1e75368_app2.docx]

**Supplemental Material 2 - Search strategy**

**Tables 1-5 describe the search terms for each database (Embase, PubMed, Web of Science Core Collection, China National Knowledge Infrastructure (CNKI), Wan Fang, and Wei Pu (VIP). A hand search of the reference lists of included studies will also be done to identify additional published studies.**

**No limit on publication date (from database inception to 20 May 2024, updated to 20 March, 2025).**

**Table 1: Search strategy - PubMed**

| **Query #** | **Query terms** |
| --- | --- |
| 1 | ((((((((((((('cardiac surgical procedur') OR ('Cardiac surgery')) OR ('cardiovascular surgery')) OR ('cardiovascular operatio*')) OR ('heart surgery')) OR ('coronary artery bypass grafting')) OR (CABG)) OR ('valv* surgery')) OR ('valv* replacement')) OR ('extracorporeal circulation')) OR ('cardiopulmonary bypass')) OR ('type A aortic dissection')) OR ('type B aortic dissection')) OR ('aortic dissection') Filters: from 1000/1/1 - 2024/5/20，updated to2025/3/20 |
| 2 | ((('risk prediction') OR ('model')) OR ('risk score')) OR ('risk assessment') Filters: from 1000/1/1 - 2024/8/12 |
| 3 | (((('Subacute Deliriums') OR ('delirium')) OR ('Postoperative delirium')) OR ('Delirium of Mixed Origin')) OR ('intensive care delirium') Filters: from 1000/1/1 - 2024/5/20，updated to2025/3/20 |
| 4 | ((#1) AND (#2)) AND (#3) Filters: from 1000/1/1 - 2024/5/20，updated to2025/3/20 |

**Table 2: Search strategy – Embase**

| **Query #** | **Query terms** |
| --- | --- |
| 1 | 'cardiac surgical procedure' OR 'cardiac surgery'/exp OR 'cardiac surgery' OR 'cardiovascular surgery'/exp OR 'cardiovascular surgery' OR 'heart surgery'/exp OR 'heart surgery' OR 'coronary artery bypass grafting'/exp OR 'coronary artery bypass grafting' OR cabg OR 'extracorporeal circulation'/exp OR 'extracorporeal circulation' OR 'type a aortic dissection'/exp OR 'type a aortic dissection' OR 'type b aortic dissection'/exp OR 'type b aortic dissection' OR 'aortic dissection'/exp OR 'aortic dissection' |
| 2 | 'risk prediction' OR 'model' OR 'risk score' OR 'risk assessment' |
| 3 | 'subacute deliriums' OR 'delirium' OR 'postoperative delirium' OR 'delirium of mixed origin' OR 'intensive care delirium' |
| 4 | #1 AND #2 AND #3 AND [12-08-1999]/sd NOT [20-03-2025]/sd |

**Table 3: Search strategy – Web of Science**

| **Query #** | **Query terms** |
| --- | --- |
| 1 | 'cardiac surgical procedure' OR 'Cardiac surgery' OR 'cardiovascular surgery' OR 'heart surgery' OR 'coronary artery bypass grafting' OR CABG OR 'extracorporeal circulation' OR 'type A aortic dissection' OR 'type B aortic dissection' OR 'aortic dissection' (Topic) |
| 2 | ‘risk prediction’ OR ‘model’ OR ‘risk score’ OR ‘risk assessment’ (Topic) |
| 3 | 'Subacute delirium' OR 'delirium' OR 'Postoperative delirium' OR 'Delirium of Mixed Origin' OR 'intensive care delirium' (Topic) |
| 4 | 1 AND 2 AND 3 |

**Table 4: Search strategy –CNKI（The following is the English translation from Chinese）**

| **Query #** | **Query terms** |
| --- | --- |
| 1 | Cardiac surgery + cardiovascular surgery + cardiothoracic surgery + postoperative cardiac surgery + open heart surgery + coronary artery bypass grafting + coronary artery bypass grafting + valve replacement + valve surgery + extracorporeal circulation + type A aortic dissection + type B aortic dissection |
| 2 | Prediction model + risk prediction + model + risk score + risk assessment + ROC curve + area under the curve |
| 3 | Postoperative delirium + postoperative cognitive dysfunction + delirium + neurological complications |
| 4 | #1 + #2 + #3 |

**Table 5: Search strategy –Wang Fang（The following is the English translation from Chinese）**

| **Query #** | **Query terms** |
| --- | --- |
| 1 | Prediction model OR Risk prediction OR model OR risk score OR risk assessment OR ROC curve OR Area under curve |
| 2 | Postoperative delirium OR postoperative cognitive dysfunction OR delirium OR neurological complications |
| 3 | Cardiac surgery OR cardiothoracic surgery OR cardiac surgery OR open heart surgery OR coronary artery bypass grafting OR coronary artery bypass graft OR valve replacement OR valve surgery OR cardiopulmonary bypass OR type A aortic dissection OR aortic dissection |
| 4 | #1 + #2 + #3 |

**Table 6: Search strategy –VIP（The following is the English translation from Chinese）**

| **Query #** | **Query terms** |
| --- | --- |
| 1 | Prediction model + risk prediction + model + risk score + risk assessment + ROC curve + area under the curve |
| 2 | Postoperative delirium + postoperative cognitive dysfunction + delirium + neurological complications |
| 3 | Cardiac surgery + cardiovascular surgery + cardiothoracic surgery + postoperative cardiac surgery + open heart surgery + coronary artery bypass grafting + coronary artery bypass grafting + valve replacement + valve surgery + extracorporeal circulation + type A aortic dissection + type B aortic dissection |
| 4 | #1 + #2 + #3 |
